# Caregiver-Patient Dynamics in Alcohol Use Disorders: A Cross-Sectional Study

**DOI:** 10.7759/cureus.91272

**Published:** 2025-08-30

**Authors:** Luciana A Ignat, Raluca O Tipa, Alina R Cehan, Vladimir C Bacârea

**Affiliations:** 1 Psychiatry, George Emil Palade University of Medicine, Pharmacy, Science, and Technology of Târgu Mureș, Târgu Mureș, ROU; 2 Psychiatry, "Prof. Dr. Alexandru Obregia" Clinical Psychiatric Hospital, Bucharest, ROU; 3 Psychiatry, Carol Davila University of Medicine and Pharmacy, Bucharest, ROU; 4 Plastic and Reconstructive Surgery, Emergency Clinical County Hospital of Târgu Mureș, Târgu Mureș, ROU; 5 Scientific Research Methodology, George Emil Palade University of Medicine, Pharmacy, Science, and Technology of Târgu Mureș, Târgu Mureș, ROU

**Keywords:** anxiety, burden, caregiver, depression, ethanol, stress, withdrawal

## Abstract

Background: Informal caregiving for individuals with alcohol use disorders (AUDs) is associated with substantial psychological and social burden. Caregivers frequently experience stress, emotional distress and reduced quality of life. However, their sociodemographic and psychological profiles are not well defined. Furthermore, the relationship between caregiver characteristics and patients’ alcohol consumption remains insufficiently explored, particularly in psychiatric inpatient settings.

Objective: This study aimed to examine the sociodemographic and psychological profiles of caregivers of male psychiatric inpatients with AUDs and to explore associations between caregiver characteristics and patients’ alcohol consumption.

Methods: A cross-sectional study was conducted between March 2023 and September 2024 at the "Prof. Dr. Alexandru Obregia" Clinical Psychiatric Hospital in Bucharest. Eighty-three caregivers of male psychiatric inpatients with AUDs were recruited during patient admissions to a specialized ward, corresponding to 83 matched patients. Caregiver data included sociodemographic characteristics and standardized assessments of depression, anxiety, stress, coping strategies and perceived burden. Patient data included sociodemographic characteristics, alcohol consumption measures and stage of change.

Results: The majority of caregivers were female (73.5%) and aged 41-50 or over 60 years, with most (78.3%) living in urban areas. High psychological distress was reported: 86.7% experienced moderate to severe depression, 98.8% anxiety and 86.8% stress. Significant associations were found between caregiver demographics and psychological indicators (p < 0.05). Patient alcohol consumption correlated significantly with caregiver depression (p = 0.005) and stress (p = 0.014), but not with anxiety, coping or burden.

Conclusion: Higher caregiver depression and stress are linked to increased patient alcohol intake. Mental health support for caregivers should be integrated into withdrawal management to improve patient outcomes.

## Introduction

Caregiving is a vital yet often overlooked aspect of global health systems, supporting the well-being of approximately 349 million people worldwide, including 18 million children and 101 million older adults [1]. In many countries, especially those with limited formal care infrastructure, informal caregivers - mostly women - bear the majority of this burden, often without sufficient training or support [2]. According to the American Psychological Association, caregivers provide essential support to individuals who are not fully independent due to age, illness, disability, or disorder, often playing key roles in both daily care and comprehensive health management [3].

A caregiver, also referred to as a “carer,” is a person who assists another individual who has limitations in functioning due to illness, disability, or age. Caregiving may involve supporting daily activities, managing medical needs, offering emotional support and ensuring safety and well-being [4]. The World Health Organization (WHO) defines caregivers broadly as “persons who provide care, paid or unpaid, to people with some degree of functional limitation, in any age group.” In health research, the term most often refers to informal caregivers, usually family members or close friends, who deliver unpaid, ongoing care outside of formal healthcare systems [5].

Alcohol use disorder (AUD) is a major global health challenge, affecting an estimated 400 million individuals aged 15 years and older in 2019, including 209 million with alcohol dependence. Beyond its direct impact on those affected, AUD generates a significant burden on families and informal caregivers [6,7].

Despite their significant contributions, caregivers face considerable emotional, physical and financial burdens, often leading to pronounced psychological distress and diminished well-being. For instance, caregivers of individuals with alcohol dependence frequently report both subjective strain and objective responsibilities that take a measurable toll on mental health [8]. In one study, nearly 79% of primary caregivers of AUD patients experienced moderate to severe burden, which correlated directly with the patients’ alcohol consumption levels [9]. A longitudinal investigation further demonstrated that when patients reduce or cease alcohol use, caregivers’ burden, depression, anxiety and stress decreased by over 50% within six weeks [10].

Caregiver fatigue can be defined as a multidimensional condition that arises from prolonged, often unplanned caregiving under overwhelming demands. It frequently develops when individuals are required to assume responsibilities that extend far beyond the typical expectations of their social or family role. For example, when spouses, children or parents must take on tasks comparable to those of professional healthcare providers, such as constant supervision, medical management or crisis intervention. These expanded responsibilities, assumed without adequate preparation or support, contribute to physical exhaustion, psychological distress and a decline in overall well-being [11,12].

Although policies to support caregivers have expanded in recent years, including services such as respite care, training and workplace protection, utilization remains low, with fewer than 20% of eligible caregivers accessing available services. Additionally, many policies lack robust evaluation mechanisms and do not adequately address issues of equity, awareness and barriers to access, particularly among vulnerable groups [13]. Beyond burden, the quality of the caregiver-receiver relationship, understood as mutuality, is recognized as a key factor affecting caregivers' well-being. Mutuality involves shared values, reciprocity, emotional closeness and respect, and has been linked to better emotional health, increased preparedness for caregiving and less burden [14,15].

Among caregivers of individuals with alcohol or substance use disorders, high levels of burden, grief, anxiety and depression have been documented, often comparable to those experienced by caregivers of individuals with severe mental illnesses such as schizophrenia [16]. Structured education and support interventions have shown effectiveness in reducing caregiver strain. Furthermore, family functioning and social support play a significant role in mediating the negative impact of care recipients’ alcohol use severity on caregiver quality of life, emphasizing potential targets for intervention [17]. Given the high prevalence of AUDs in the European region, where 1 in 11 adults is affected and alcohol-attributable mortality rates are nearly three times the global average, understanding the dynamics of caregiver burden, grief and well-being in this context is critical [18].

The Transtheoretical Model of Change (TTM), developed by Prochaska, DiClemente and Norcross, is a widely applied framework for understanding intentional behavior change, particularly in the context of substance use disorders. Also known as the “Stages of Change” model, it conceptualizes change as a dynamic and cyclical process, progressing through five stages: precontemplation (no intention to change), contemplation (awareness of the problem but ambivalence toward change), preparation (intention and initial steps toward change), action (active modification of behavior) and maintenance (sustaining new behaviors and preventing relapse). By recognizing that individuals vary in their readiness to change, the model emphasizes the importance of tailoring interventions to the person’s motivational stage, thereby enhancing clinical effectiveness and engagement [19,20].

Over the past decades, TTM has gained widespread acceptance in clinical and research settings due to its flexibility and practical utility across a range of behavioral health interventions, including alcohol misuse, smoking cessation and lifestyle modification [20]. In the present study, readiness to change was measured using the Readiness to Change Questionnaire (RCQ), which operationalizes the TTM framework in the context of AUDs, ensuring both theoretical grounding and clinical relevance.

AUD is a medical condition characterized by the inability to control or stop alcohol consumption, despite facing social, occupational or health-related consequences. Individuals with AUD experience compulsive drinking, develop tolerance and may suffer withdrawal symptoms when they are not drinking. Also, they often continue to drink even when it causes harm. Diagnosis of AUD is based on specific criteria outlined in the Diagnostic and Statistical Manual of Mental Disorders, Fifth Edition (DSM-5), with severity ranging from mild to severe, depending on the number of symptoms present [21].

AUDs impact not only individuals with dependence but also their caregivers. Informal caregivers often face significant psychological stress, including anxiety, depression and perceived burden, which can be influenced by sociodemographic factors and caregiving demands. Additionally, caregiver well-being and psychosocial functioning can impact patient outcomes, such as daily alcohol consumption levels. The patient’s willingness to change, a key component in substance use treatment models, may also be related to drinking behaviors, influencing both treatment engagement and long-term recovery [22]. Exploring the links between caregiver characteristics, psychological health and patient drinking patterns is crucial for understanding the caregiver-patient relationship in AUD cases. This knowledge can guide the development of comprehensive, family-centered strategies to improve outcomes for both caregivers and patients.

This study aims to examine the socio-demographic and psychological characteristics of caregivers of patients admitted for ethanol withdrawal, with a focus on their emotional distress, coping strategies and perceived burden. It further seeks to explore how these caregiver traits relate to patients’ readiness for change and alcohol use, in order to identify factors that may inform targeted interventions to support caregiver well-being in the context of AUDs.

Hypotheses

H1

Caregivers’ sociodemographic characteristics (gender, educational level, relationship to the patient and time spent caregiving) are associated with their psychological outcomes, with higher distress observed among female caregivers, those with lower education and those providing more intensive care [23].

H2

Being at a more advanced stage of behavioral change (action) is associated with lower daily alcohol intake compared to earlier stages (precontemplation or contemplation), consistent with the TTM.

H3

Caregivers’ psychological outcomes (stress, anxiety, depression, coping and perceived burden) are related to patients’ daily alcohol consumption, with more adverse outcomes observed among caregivers of patients reporting higher intake.

H4

The duration of caregiving is associated with the patients’ motivational stage of change, according to TTM.

H5

Caregivers’ coping levels are associated with the patients’ motivational stage of change, according to TTM.

## Materials and methods

This observational, cross-sectional study was conducted at the "Prof. Dr. Alexandru Obregia" Clinical Psychiatric Hospital in Bucharest. The sample comprised 83 caregivers and the 83 male patients under their care, who were admitted to the ward for the management of ethanol withdrawal. Patients were enrolled consecutively, indicating that all eligible individuals presenting during the study period were included in the order of admission, without preselection. Data collection was carried out between March 2023 and September 2024.

Participants were informal (unpaid) caregivers of patients whose primary admission diagnosis, established within 24-48 hours of hospitalization according to the International Classification of Diseases, Tenth Revision (ICD-10) criteria, was either F10.3 (mental and behavioral disorders due to alcohol use - withdrawal state) or F10.4 (mental and behavioral disorders due to alcohol use - withdrawal state with delirium).

Inclusion and exclusion criteria for patients

Eligible patients were aged 18 years or older, literate in Romanian, and had a primary admission diagnosis established within 24-48 hours of hospitalization according to the ICD-10 classification: F10.3 - Mental and behavioral disorders due to alcohol use: withdrawal state, and/or F10.4 - Mental and behavioral disorders due to alcohol use: withdrawal state with delirium. Written informed consent to participate was obtained after remission of alcohol withdrawal symptoms, ensuring decision-making capacity.

Exclusion criteria for patients included severe psychiatric comorbidities or cognitive impairments that could interfere with the completion of study assessments. Specifically, patients with any of the following ICD-10 diagnoses were excluded: F20 - Schizophrenia; F22 - Persistent delusional disorders; F23 - Acute and transient psychotic disorders; F25 - Schizoaffective disorders; F28 - Other nonorganic psychotic disorders; F29 - Unspecified nonorganic psychosis; F31 - Bipolar affective disorder; F32.2 - Major depressive episode, severe without psychotic symptoms; F32.3 - Major depressive episode, severe with psychotic symptoms; F33.2 - Recurrent depressive disorder, current episode severe without psychotic symptoms; F33.3 - Recurrent depressive disorder, current episode severe with psychotic symptoms; F70-F73 - Intellectual disabilities; and F01-F03 - Severe cognitive impairments.

Inclusion and exclusion criteria for caregivers

Caregivers were eligible if they were informal (unpaid) caregivers, aged 18 years or older, identified as the primary caregiver of the patient, capable of providing informed consent, and literate in Romanian to ensure accurate completion of standardized self-report instruments. The patients under their care had a confirmed ethanol withdrawal diagnosis documented in the medical record by a licensed psychiatrist during the actual admission.

Exclusion criteria for caregivers were applied to minimize confounding and ensure data quality. Caregivers were excluded if they self-reported or had documented histories of severe psychiatric disorders (schizophrenia spectrum disorders, bipolar disorder type I or II, major depressive disorder with psychotic features, or intellectual disabilities). Additional exclusion criteria: current substance use disorder (excluding nicotine), significant cognitive impairment or neurological conditions limiting informed consent or study participation. Paid/professional caregivers were excluded.

**Figure 1:**
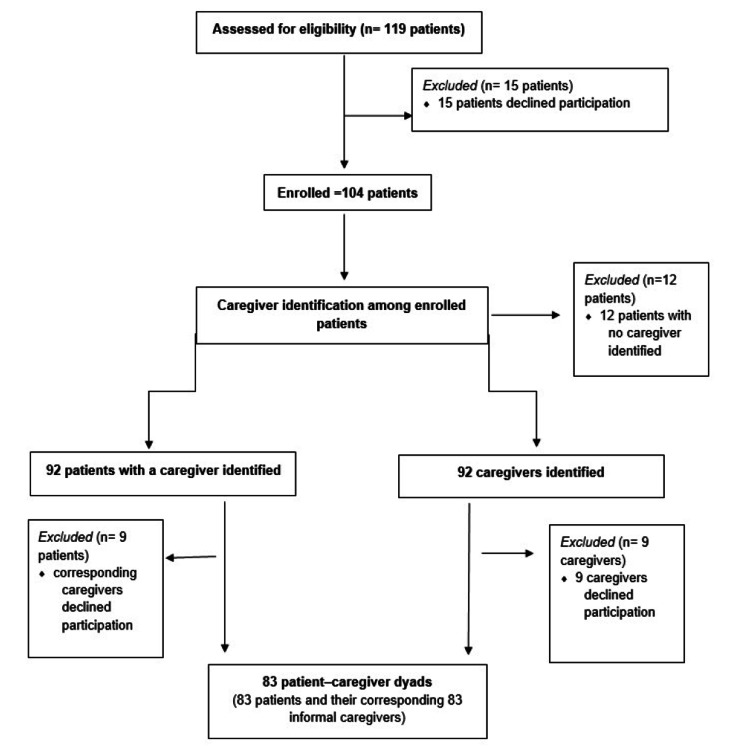
Flow of patient and caregiver inclusion in the study

A total of 119 patients were assessed for eligibility. Of these, 15 declined participation, resulting in 104 patients initially enrolled. Caregivers were identified for 92 of these patients, while 12 patients did not have an identifiable primary caregiver. Of the 92 eligible caregivers, nine declined participation. The final analytic sample comprised 83 patient-caregiver dyads (83 patients and their corresponding 83 informal caregivers), as presented in Figure 1.

All eligible caregivers were informed about the study's objectives and procedures. Written informed consent was obtained before participation. Data collection involved completing standardized self-report measures assessing depression, anxiety, stress, coping strategies, perceived caregiver burden and demographic variables. For the patients, socio-demographic information and the average number of alcoholic drinks consumed per day were collected from clinical observation records. After the resolution of acute withdrawal symptoms, patients were asked to complete the RCQ, a validated tool often used in addiction research.

The study protocol was reviewed and approved by the local ethics committee of the "Prof. Dr. Alexandru Obregia" Clinical Psychiatric Hospital (No. 109/12.01.2023) on January 12, 2023, for studies involving humans. Participation was voluntary and anonymous. All data were treated confidentially, consistent with national and international ethical guidelines governing research involving human participants.

Instruments

All psychological instruments used in this study were selected based on their established psychometric validity and accessibility for academic research. Throughout the study, all copyright and intellectual property rights were fully respected, following international ethical guidelines governing the use of proprietary psychological assessment tools in research.

The Depression Anxiety Stress Scales - 21 items (DASS-21)

DASS-21, developed by Lovibond and Lovibond (1995), is a public domain instrument designed to assess the severity of depression, anxiety and stress symptoms over the past week. It consists of 21 items rated on a 4-point Likert scale, with subscale scores multiplied by 2 to match the full 42-item version. The Romanian version, validated by Cognitrom, has demonstrated strong internal consistency (depression α = 0.85; anxiety α = 0.80; stress α = 0.86) and robust construct and discriminant validity. As it is unrestricted in use, no license was required for its administration in this study [24].

The Brief Resilient Coping Scale (BRCS)

BRCS, developed by Sinclair and Wallston (2004), is a four-item self-report instrument designed to assess an individual’s tendency to cope with stress in adaptive, resilient ways. Each item is rated on a 5-point Likert scale, with higher total scores indicating greater resilient coping. The BRCS has demonstrated acceptable internal consistency (Cronbach’s α = 0.70-0.76) and good construct validity in relation to stress, coping and psychological well-being. In this study, the BRCS was used in its original format and in Romanian, based on the authors’ explicit statement allowing unrestricted use for academic, non-commercial research purposes. Public availability of the scale is further supported by its inclusion on platforms such as PsyToolkit. No modifications or licensing were required, and use adhered to ethical standards and intellectual property guidelines. It is important to note that Mapi Research Trust does not distribute this clinical outcome assessment (COA) on behalf of its developer, and no formal licensing from Mapi was required or applicable for the use of this instrument [25].

The Zarit Burden Interview - 12-Item Version (ZBI-12)

ZBI-12, developed by Zarit et al. (1980), is a widely used self-report instrument for assessing the subjective burden experienced by informal caregivers. Created initially for dementia caregiving contexts, it has been validated for use with caregivers of individuals with various chronic and psychiatric conditions. The ZBI-12 uses a 5-point Likert scale (0 = “never” to 4 = “nearly always”), with total scores ranging from 0 to 48, where higher scores reflect greater caregiver burden. The scale demonstrates strong psychometric properties, with Cronbach’s alpha typically ranging from 0.83 to 0.89. In this study, the licensed Romanian version (α = 0.87) was used under formal authorization from the Mapi Research Trust (License No. 75580). No adaptations or digitizations were made, and full compliance with licensing and intellectual property requirements was maintained [26].

The Readiness to Change Questionnaire (RCQ)

RCQ is a 12-item self-report instrument developed to assess an individual’s motivational stage of change regarding problematic substance use, based on the Transtheoretical Model of Behavior Change. Items are rated on a 5-point Likert scale and assess cognitive and motivational components of change. In this study, the RCQ was administered to patients following the resolution of acute withdrawal symptoms associated with ethanol dependence, to assess motivational readiness and guide individualized treatment planning. The Romanian version used was previously published in *Mental Health Measurement: A Compendium of Scales and Interviews Used in the Assessment of Psychopathological Disorders* (Vraști, R.; www.vrasti.org). The author granted explicit permission for its use via email correspondence dated 16 August 2022. Consequently, the instrument was used with full authorization for non-commercial academic research purposes, and no licensing agreement was required [27].

Statistical analysis

Descriptive statistics were used to summarize the sociodemographic data of caregivers and patients, including frequencies, percentages, means, standard deviations, medians, skewness and kurtosis. Normality of continuous variables was assessed using the Kolmogorov-Smirnov and Shapiro-Wilk tests.

Associations between caregiver sociodemographic variables and psychological indicators (depression, anxiety, stress, coping and perceived burden) were evaluated using chi-square tests, with results reported as p-values and contingency coefficients.

The psychosocial burden of caregivers was assessed during the patients’ current admission for ethanol withdrawal, once acute withdrawal symptoms had stabilized. This timing ensured that the burden measured reflected the immediate context of hospitalization, while also being influenced by the cumulative experience of long-term caregiving.

In addition, we recorded the duration of caregiving, expressed as the number of years the caregiver had been caring for the patient. To explore the relationship between caregiving duration and the patients’ motivational stage of change, caregiving time was grouped into ranges (<5 years, 5-10 years, 11-15 years, 16-20 years, and >20 years). Chi-square tests of independence were performed to examine whether the length of caregiving was associated with the patients’ stage of change according to the Transtheoretical Model. We also examined the caregivers’ coping level, categorized as low or medium, in relation to the patients’ stage of change, using the same statistical approach.

Because daily alcohol consumption was non-normally distributed (confirmed by normality tests; skewness = 2.42, kurtosis = 8.43), non-parametric tests were applied. Kruskal-Wallis tests were used to examine group differences in alcohol consumption across caregiver psychological categories and patient stage of change, with post-hoc Dunn-Bonferroni tests conducted for pairwise comparisons. Mann-Whitney U tests were employed for two-group comparisons (stress, anxiety, coping and burden levels), and effect sizes (r) were reported where applicable. Data were analyzed using IBM SPSS Statistics for Windows, Version 29 (Released 2021; IBM Corp., Armonk, New York, United States) and RStudio version 2024.12.0+467 (RStudio Team 2015, RStudio: Integrated Development for R. RStudio, Inc., Boston, MA). The significance level was set at p < 0.05 for all statistical analyses.

## Results

The study included 83 caregivers, predominantly female (73.5%), with the majority aged 41-50 years (33.7%) or over 60 years (27.7%). A total of 78.3% resided in urban areas, and 61.5% were married. Educational attainment was highest for post-secondary vocational training (42.2%), followed by primary education (25.3%) and high school (24.1%). Most caregivers were the patient’s wife (38.6%), sister (18.1%) or mother (14.5%). The duration of caregiving varied, with 32.5% providing care for less than five years and 25.3% for over 20 years. Regarding psychological measures, moderate or severe depression was reported by 86.7% of caregivers, moderate or severe anxiety by 98.8% and severe stress by 86.8%. Low coping capacity was observed in 55.4% of caregivers, and a high perceived burden was reported in 65.1% of them. The entire dataset is summarized in Table 1.

**Table 1:** Sociodemographic profile of caregivers in the study sample

Variable	Category	n (%)
Gender	Female	61 (73.49)
	Male	22 (26.51)
Age	Under 25 years	1 (1.20)
	26-30 years	4 (4.82)
	31-40 years	17 (20.48)
	41-50 years	28 (33.73)
	51-60 years	10 (12.05)
	Over 60 years	23 (27.71)
Area of residence	Urban	65 (78.31)
	Rural	18 (21.69)
Education level	Primary (1-8 grades)	21 (25.30)
	High school	20 (24.10)
	Post-secondary (vocational)	35 (42.17)
	Higher education (university)	7 (8.43)
Socio-occupational status	Employed	47 (56.63)
	Retired	24 (28.92)
	Unemployed	12 (14.46)
Marital status	Married	51 (61.45)
	Single	15 (18.07)
	Widowed	14 (16.87)
	Divorced	3 (3.61)
Duration of caregiving/co-residence	Less than 5 years	27 (32.53)
	5-10 years	20 (24.10)
	11-15 years	9 (10.84)
	16-20 years	6 (7.23)
	More than 20 years	21 (25.30)
Relationship with the patient	Wife	32 (38.55)
	Sister	15 (18.07)
	Mother	12 (14.46)
	Father	9 (10.84)
	Child	5 (6.02)
	No family relation	10 (12.05)

Analysis revealed significant associations between caregivers’ sociodemographic factors and psychological outcomes. Gender was significantly associated with depression, anxiety, coping ability and perceived burden (all p < 0.05), with female caregivers showing greater emotional vulnerability; no association was found with stress. The degree of kinship showed strong, significant associations with all psychological outcomes (all p < 0.001). The duration of caregiving was significantly associated with depression, stress, coping and perceived burden, but not anxiety. These findings highlight the importance of relational and contextual caregiving factors, particularly kinship and caregiving duration, in predicting psychological distress and perceived burden among caregivers.

Coping was assessed using the BRCS, where higher scores reflect greater coping capacity (low: ≤13, medium: 14-16, high: ≥17). Caregivers in this sample reported only low and medium coping levels. Gender was significantly associated with coping ability, with female caregivers reporting lower coping scores than male caregivers (p < 0.05). Kinship also showed strong associations with coping ability (p < 0.001). A complete overview of the data is presented in Table 2.

**Table 2:** Associations between sociodemographic variables and psychological indicators in caregivers All values are reported as p-value/contingency coefficient. P < 0.05 indicates statistical significance. The contingency coefficient indicates the strength of association between categorical variables (higher values = stronger association).

Variable	Depression (p-value/contingency coefficient)		Anxiety (p-value/contingency coefficient)		Stress (p-value/contingency coefficient)		Coping scale (p-value/contingency coefficient)		Perceived burden (p-value/contingency coefficient)	
Gender	<0.001	0.79	<0.001	0.65	0.258	0.18	<0.001	0.75	0.031	0.33
Area of residence	0.722	0.13	0.713	0.06	0.015	0.37	<0.001	0.72	<0.001	0.60
Level of education	0.002	0.55	0.476	0.24	0.040	0.43	<0.001	0.61	<0.001	0.67
Socio-professional category	0.074	0.38	0.013	0.44	0.054	0.36	0.070	0.35	0.005	0.48
Degree of kinship	<0.001	0.733	<0.001	0.525	<0.001	0.601	<0.001	0.464	<0.001	0.810
Time spent caring/living with patients	<0.001	0.535	0.628	0.176	<0.001	0.473	<0.001	0.482	0.003	0.570

The study sample comprised 83 male patients, reflecting the gender-specific admission profile of the psychiatric ward. Over half (54.2%) were aged 41-50 years, followed by those aged 31-40 (22.9%) and 51-60 (18.1%). Marital status was nearly evenly distributed among single individuals (33.7%), married individuals (33.7%) and divorced individuals (31.3%). The socio-demographic characteristics of the patients are summarized in Table 3. Daily alcohol consumption exhibited substantial positive skewness (skewness = 2.42) and leptokurtosis (kurtosis = 8.43), with a mean of 18.9 drinks per day (SD = 11.4), a median of 17 and a range of 6-70. Tests of normality (Kolmogorov-Smirnov and Shapiro-Wilk) all indicated significant deviations from normality (p < 0.05).

**Table 3:** Sociodemographic profile of patients in the study sample

Variable	Category	n (%)
Age	26-30	2 (2.41)
	31-40	19 (22.89)
	41-50	45 (54.22)
	51-60	15 (18.07)
	Over 60	2 (2.41)
Area of residence	Urban	57 (68.67)
	Rural	26 (31.33)
Marital status	Single	28 (33.73)
	Married	28 (33.73)
	Divorced	26 (31.33)
	Widowed	1 (1.20)
Level of education	Primary (1-8 grades)	17 (20.48)
	High school	30 (36.14)
	Post-secondary (vocational)	19 (22.89)
	Higher education (university)	17 (20.48)
Socio-occupational category	Employed with a work contract	47 (56.63)
	Retired due to medical disability	14 (16.87)
	Unemployed	11 (13.25)
	Day laborer	8 (9.64)
	Retired	3 (3.61)

The association between stage of change and daily alcohol consumption is summarized in Table 4. A Kruskal-Wallis H test revealed a significant association between patients’ stage of change and daily alcohol consumption, χ^2^(2) = 8.41, p = 0.015. Post-hoc comparisons are presented in Table 4. Pairwise differences were not statistically significant after adjustment.

**Table 4:** Dunn's post-hoc comparisons of daily alcohol consumption by stage of change

Pairwise comparison	Adjusted p (Bonferroni)	Interpretation
Contemplation vs. action	0.093	Approached significance
Contemplation vs. precontemplation	0.090	Approached significance
Action vs. precontemplation	1.000	Not significant

Associations between caregiver psychological factors and patient alcohol consumption are summarized in Table 5. Non-parametric analyses revealed a significant association between caregiver depression levels and patients’ daily alcohol consumption (p = 0.005). Post-hoc tests showed patients with caregivers reporting severe depression consumed significantly more alcohol than those with moderately depressed caregivers (p = 0.004). Caregiver stress was also significantly associated with patient alcohol use (p = 0.014, r = 0.24), with higher stress linked to greater consumption. No significant associations were found between caregiver anxiety (p = 0.522), coping ability (p = 0.151) or perceived burden (p = 0.424) and patient alcohol intake.

**Table 5:** Association between caregivers’ psychological characteristics and patients’ daily alcohol consumption

Caregiver variable	Comparison groups	Statistical test	P-value	Effect size (r/coef.)
Depression	Mild, moderate, severe	Kruskal-Wallis	0.005	-
	Moderate vs. severe	Dunn-Bonferroni	0.004	-
	Mild vs. moderate/mild vs. severe	Dunn-Bonferroni	>0.05	-
Anxiety	Severe vs. moderate	Mann-Whitney U	0.522	r = 0.01
Stress	Severe vs. moderate	Mann-Whitney U	0.014	r = 0.24
Coping	Low vs. medium	Mann-Whitney U	0.151	r = 0.11
Perceived burden	High vs. medium	Mann-Whitney U	0.424	r = 0.02

The association between the duration of caregiving and the patient’s stage of change, as defined by the Transtheoretical Model, was examined using a chi-square test of independence. The analysis revealed a statistically significant relationship (p = 0.028). The contingency coefficient (C = 0.41) further suggested that the strength of this association was moderate. This finding suggests that the psychosocial burden of caregivers, as reflected in the length of caregiving, may be related to the motivational stage of change of patients. Specifically, caregivers with longer caregiving experience (>20 years) were more frequently associated with patients in the contemplation stage. In contrast, those with intermediate caregiving durations (5-20 years) demonstrated a more heterogeneous distribution across the action, contemplation and precontemplation stages. By contrast, the association between caregivers’ coping level and the patients’ stage of change was not statistically significant (p = 0.387). While caregivers with medium coping strategies appeared somewhat more often in relation to patients in the action stage, this tendency did not reach statistical significance. Chi-square tests examining associations between caregiving characteristics and patients’ stage of change are summarized in Table 6.

**Table 6:** Summary of chi-square tests examining associations between caregiving characteristics and patients’ stage of change

Caregiving variable	P-value	Contingency coefficient (C)
Duration of caregiving	0.028	0.41
Coping level (low vs. medium)	0.387	0.15

## Discussion

A total of 83 informal caregivers participated in this study, most of whom were female and primarily aged 41-50 or over 60 years. The findings indicate that the duration of caregiving is significantly associated with the patient’s motivational stage of change, with long-term caregiving (>20 years) more frequently linked to patients in the contemplation stage. This pattern may reflect the accumulated psychosocial burden and the challenges of sustaining motivation for change across repeated relapse-remission cycles. In contrast, no significant relationship was found between coping level (low vs. medium) and the patient’s stage of change, suggesting that caregivers’ coping strategies, at least as captured by this measure, may not directly influence or align with patients’ motivational readiness.

Despite the absence of statistical significance, coping responses remain clinically relevant. They shape the quality of the caregiving relationship and the overall well-being of both caregivers and patients. Integrating caregiver-focused support within intervention planning may therefore enhance treatment outcomes and strengthen family resilience [12].

Nearly 87% of caregivers met criteria for moderate or severe depression, 99% for anxiety and 87% for severe stress; more than half reported low coping ability (55.4%) and high perceived burden (65.1%). These findings align with previous studies indicating that caregivers of individuals with AUD experience mental health challenges similar to those of caregivers of patients with severe mental illnesses like schizophrenia, and these challenges are notably greater than those faced by non-caregivers [16].

Furthermore, research indicates that female caregivers and those with less education are especially susceptible to psychological distress in AUD settings, as women often assume caregiving roles that carry strong emotional and social expectations, which can intensify symptoms of depression and anxiety [10]. This study's data also aligns with international findings showing that caregivers of AUD patients frequently experience higher levels of stress, anxiety and depression compared with caregivers of individuals with other conditions [28]. These high stress levels may stem from the ongoing and unpredictable nature of alcohol dependence, as well as the stigma, financial difficulties and interpersonal challenges associated with the condition, all of which are recognized predictors of caregiver burnout. Prolonged caregiving responsibilities and complex family dynamics can further intensify these effects, particularly in cultures where informal and unpaid caregiving is undervalued [29].

Significant associations emerged between caregivers’ sociodemographic profiles and their psychological outcomes. Greater levels of emotional strain, including depression and anxiety, were observed among female caregivers, individuals with lower educational attainment and those residing in rural areas. Closer kinship relationships and extended caregiving duration were also linked to elevated stress, reduced coping ability and heightened perceived burden. In particular, female caregivers reported higher levels of depression, anxiety, lower coping capacity and greater perceived burden, consistent with prior studies demonstrating that women in caregiving roles are especially vulnerable to psychological distress due to cultural, social and emotional expectations [30].

Area of origin is also associated with stress, coping and perceived burden. Similar patterns have been documented in studies comparing urban and rural caregivers, where urban caregivers often report higher stress and burden due to limited community cohesion and reduced access to informal support networks [31]. Educational status showed significant associations with depression, stress, coping and perceived burden in our sample, consistent with prior research indicating that lower education may limit caregivers’ ability to navigate health systems, mobilize resources and employ adaptive coping strategies, thereby heightening vulnerability to mental health difficulties [32].

The results of this study indicate that both the degree of kinship and the duration of caregiving were significantly associated with all assessed psychological outcomes. This aligns with previous research in the contexts of dementia, palliative care and chronic illness, which consistently demonstrate that closer familial relationships, particularly among spouses, along with extended caregiving, are key factors that predict higher caregiver burden, as well as increased levels of depression, anxiety and stress [33]. Notably, caregiver anxiety did not significantly differ by area of origin, education or caregiving duration, suggesting that anxiety may be influenced by more complex or individual-level factors not captured in this study.

The exclusively male patient sample, predominantly aged 41-50 years and primarily residing in urban areas (68.7%), reflects demographic trends commonly observed in clinical populations undergoing alcohol withdrawal. Participants represented a range of educational and employment backgrounds. The mean alcohol intake of 18.9 standard drinks per day is consistent with established patterns in individuals with alcohol dependence, which typically show non-normal distributions due to a subset of individuals with particularly harmful use [34].

This skewed distribution underscores heterogeneity in consumption severity, with implications for screening and intervention: a large subset presents as heavy drinkers, necessitating intensive treatment, while a considerable part still consumes moderately. Variability may obscure group-level effects in traditional parametric analyses, which justifies our use of non-parametric statistics. In similar inpatient cohorts, average drinking often clusters around 15-20 drinks/day, with skewness and leptokurtosis linked to greater withdrawal severity and complications [35].

The male-only composition of our sample aligns with evidence that men exhibit higher rates of AUD and are more frequently hospitalized for withdrawal. Prior studies also show gender-specific comorbidity patterns, with men more often diagnosed with substance use disorders and antisocial personality disorders, while women more frequently present with mood and anxiety disorders and mention stigma as a treatment barrier. Men additionally tend to meet more diagnostic criteria, experience longer episodes and initiate drinking at a younger age, whereas women are more likely to report a family history of AUDs [36]. While this gender homogeneity improves internal consistency, it inevitably limits generalizability to female patients. Our data support existing literature at multiple levels: demographic profile aligns with epidemiological trends in male alcohol dependence, highly skewed consumption echoes clinical distributions documented in withdrawal cohorts and non-normal distribution reinforces methodological decisions for using robust statistical tests [37].

This study identified a significant association between patients’ stage of change and their daily alcohol consumption, with individuals in the contemplation stage reporting higher levels of intake. Although post-hoc comparisons did not remain statistically significant after correction, likely due to small and uneven group sizes, these findings are consistent with existing literature indicating that individuals in the contemplation stage often acknowledge the need for change but have not yet initiated behavioral modification, thereby maintaining patterns of harmful alcohol use [38]. The lack of significant differences between other stages is consistent with previous studies that have reported difficulties in detecting stage-based variations in alcohol consumption within small samples [39]. From a clinical perspective, these findings underscore the importance of implementing motivational interventions specifically tailored to individuals in the contemplation stage, as such approaches have been shown to support progression toward the action stage and contribute to reductions in alcohol use [40].

This study identified significant associations between caregiver mental health and patients’ daily alcohol consumption. Patients whose caregivers reported severe depressive symptoms consumed substantially more alcohol than those whose caregivers exhibited moderate depression, suggesting a possible influence of caregiver mental health on patient drinking behavior. Similarly, higher caregiver stress was linked to greater alcohol intake among patients, consistent with prior research showing that caregiver distress can exacerbate maladaptive behaviors in care recipients, including substance use [41].

Previous studies have highlighted the cyclical relationship between caregiver psychological burden and patient substance use outcomes. Caregivers experiencing high levels of stress and depression often have reduced emotional and practical support capacity, which can undermine patient recovery efforts and increase relapse risk [42]. Caregiver mental health has also been linked to adverse patient outcomes across various chronic conditions, including mental health and substance use disorders, underscoring the critical role of caregiver well-being in delivering effective and comprehensive care [43,44].

In contrast, caregiver anxiety, coping ability and perceived burden were not significantly associated with patient alcohol consumption. Similar results have been reported in previous studies, suggesting that while general burden and anxiety contribute to overall caregiver distress, depression and stress may have a more direct impact on patient drinking behaviors [45,46].

These findings highlight the importance of incorporating caregiver mental health support into comprehensive treatment programs for AUD. Interventions aimed at reducing caregiver depression and stress, such as psychoeducation, psychological counseling and structured social support services, may yield downstream benefits by contributing to decreased patient alcohol consumption and enhanced recovery outcomes. In many settings, such support includes family therapy, respite care, mutual-help groups like Alcoholics Anonymous and community-based programs that provide stress management and coping skills training. Governmental policies also play a critical role; for example, the WHO’s Global Strategy to Reduce the Harmful Use of Alcohol emphasizes the integration of family and community support into national alcohol policies [40]. Some countries have introduced caregiver support allowances, workplace protections and publicly funded counseling programs to mitigate the psychological burden of caregiving. Expanding these initiatives and ensuring access to affordable mental health services can strengthen caregiver resilience while also improving recovery outcomes for individuals with AUD [47].

Limitations

This study has several limitations that should be acknowledged. The cross-sectional design prohibits causal inferences from the observed associations, and the relatively small and uneven subgroup sizes may have limited statistical power. Reliance on self-report measures may have introduced response biases, such as underreporting of alcohol use or overreporting of coping strategies, which could have affected the accuracy of the results. In addition, categorizing continuous psychological variables may have reduced the sensitivity of the analyses. Finally, because the study focused on a specific inpatient population, the findings may not be fully generalizable to broader clinical or community samples.

Future research should address these limitations by employing longitudinal designs that can clarify causal pathways between caregiver distress and patient outcomes. Larger and more balanced samples are needed to improve statistical power and enable more precise subgroup analyses. Extending research into outpatient and community-based populations will also be essential for enhancing generalizability and for informing the development of targeted, family-centered interventions.

## Conclusions

This study highlights the associations between caregiver characteristics and psychological distress, showing that higher levels of caregiver depression and stress were linked to increased patient alcohol consumption, particularly among individuals in the contemplation stage of change. Although these findings suggest a potential connection between caregiver well-being and patient behavior in the context of AUD, the direction and underlying mechanisms of this relationship remain uncertain. The results should therefore be interpreted as indicative rather than conclusive, underscoring the importance of integrated, family-centered treatment approaches. Such approaches may be enhanced by incorporating structured caregiver assessments into routine care, expanding access to psychoeducation and counseling, and tailoring support to sociodemographic vulnerabilities, thereby strengthening caregiver resilience and improving recovery outcomes for patients.
